# Comparison of clinical profiles and care for patients with incident versus recurrent acute coronary syndromes in France: Data from the MONICA registries

**DOI:** 10.1371/journal.pone.0263589

**Published:** 2022-02-14

**Authors:** Suzanne Machta, Victoria Gauthier, Jean Ferrières, Michèle Montaye, Samantha Huo Yung Kai, Stefy Gbokou, Katia Biasch, Marie Moitry, Philippe Amouyel, Jean Dallongeville, Aline Meirhaeghe

**Affiliations:** 1 Univ. Lille, Inserm, CHU Lille, Institut Pasteur de Lille, U1167 - RID-AGE - Facteurs de risque et déterminants moléculaires des maladies liées au vieillissement, Lille, France; 2 Department of Cardiology, Toulouse Rangueil University Hospital, Toulouse, France; 3 Inserm UMR 1295, CERPOP, Université de Toulouse III Paul Sabatier, Toulouse, France; 4 Department of Epidemiology, Health Economics and Public Health, INSERM UMR 1027, Toulouse, France; 5 Department of Epidemiology, Toulouse University Hospital, Toulouse, France; 6 Department of Epidemiology and Public Health, University of Strasbourg, Strasbourg, France; 7 Department of Public Health, University Hospital of Strasbourg, Strasbourg, France; University of Bologna, ITALY

## Abstract

**Background:**

Recurrence is common after an acute coronary syndrome (ACS). In order to better assess the prognosis for patients with ACS, we compared clinical profiles, treatments, and case fatality rates for incident vs. recurrent ACS.

**Methods:**

We enrolled 1,459 men and women (age: 35–74) living in three geographical areas covered by French MONICA registries and who had been admitted to hospital for an ACS in 2015/2016. We recorded and compared the clinical characteristics and medical care for patients with an incident vs. a recurrent ACS.

**Results:**

Overall, 431 (30%) had a recurrent ACS. Relative to patients with an incident ACS, patients with recurrence were older (p<0.0001), had a greater frequency of NSTEMI or UA (p<0.0001), were less likely to show typical symptoms (p = 0.045), were more likely to have an altered LVEF (p<0.0001) and co-morbidities. Angioplasty was less frequently performed among patients with recurrent than incident NSTEMI (p<0.05). There were no intergroup differences in the prescription of the recommended secondary prevention measures upon hospital discharge, except for functional rehabilitation more frequently prescribed among incident patients (p<0.0001). Although the crude 1-year mortality rate was higher for recurrent cases (14%) than for incident cases (8%) (p<0.05), this difference was no longer significant after adjustment for age, sex, region, diagnosis category and LVEF.

**Conclusion:**

Compared with incident patients, recurrent cases were more likely to have co-morbidities and to have suboptimal treatments prior to hospital stay, reinforcing the need for secondary prevention.

## Introduction

Between 12% and 42% of patients hospitalized for an acute coronary syndrome (ACS) will have experienced a coronary event previously [1–6]. The few studies to have compared patients with incident vs. recurrent ACS evidenced marked differences in clinical profiles, management, and prognosis [3–10]. Compared with patients hospitalized for an incident event, patients hospitalized for a recurrent ACS generally have higher prevalences of diabetes, hyperlipidemia, hypertension and vascular disease and thus a worse prognosis [3–10].

Most published studies have compared incident and recurrent cases with regard to risk factors, acute-phase clinical care, and mortality rates [1–10]. However, very few studies have explored drug treatments before and after hospital admission and other types of medical care (revascularization, functional rehabilitation etc…) in this setting [2,5,8,10]. Furthermore, there were interstudy differences in the inclusion criteria (e.g. ST-segment elevation myocardial infarction (STEMI) only) and data sources (hospital databases, cohort studies, clinical trials, and population-based registries) [7–9,11].

The goal of the present study was to compare incident and recurrent cases of ACS with regard to clinical characteristics and care. More precisely, we compared (i) the distribution of the different types of ACS, (ii) the patients’ cardiovascular risk factor profiles and their treatments before the acute event, (iii) the care received during the acute phase of the event and at discharge, and (iv) survival 28 days and 1 year after the event. To this end, we analyzed data from the French MONICA population-based registries.

## Methods

### Population

The study population comprised all men and women aged 35–74 and who had been hospitalized for an ACS between October 1^st^, 2015, and March 31^st^, 2016. All the included patients resided in one of the three distinct geographical areas covered by the MONICA registries, each of which covers a population of about one million inhabitants: the Lille urban area in northern France, the Bas-Rhin county in eastern France, and the Haute-Garonne county in south-western France [12]. The registries are part of a national network of registries depending of *Santé Publique France*, the national agency of epidemiological surveillance. The methodology is validated and approved by Santé Publique France and used for national statistics.

The MONICA registries cover all public and private hospitals (including emergency departments) and all hospitalized patients—regardless of the admission department. Multiple sources were cross-checked to ensure exhaustive data collection: discharge letters, computerized lists containing the diagnosis stated upon hospital discharge, emergency department computer lists, death certificates, etc… The documentation of ACS was based on the patient’s clinical history and hospital records. Clinical diagnoses were obtained from medical records. To be included in the study, patients had to have been hospitalized with one of the following clinical diagnoses: acute myocardial infarction (AMI), acute coronary syndrome (ACS) or unstable angina (UA). Coronary deaths were also included. Sudden deaths were excluded because clinical, laboratory and electrocardiographic data were often missing. Other nonfatal coronary episodes (such as decompensations of pre-existing coronary heart diseases) and ACS in already hospitalized patients (for another disease than ACS) were also excluded, in order to reduce heterogeneity. Multiple events within a 28-day period were treated as a single event [13]. An incident case was defined as an ACS occurring in a patient with no history of acute or chronic coronary heart disease. A recurrent case was defined as an ACS occurring in a patient with a history of coronary heart disease, at least 28 days after their incident event [14].

### Ethical approval

The study was approved by the Advisory Committee on Data Processing in Health Research (Co*mité Consultatif sur le Traitement de L’information en Matière de Recherche dans le domaine de la Santé*; reference: 97002.A), the French National Data Protection Commission (*Commission Nationale de l’Informatique et des Libertés*; reference: 986001v3) and the French National Registry Committee (reference: 2016/11/9). The need for individual consent was waived by the ethics committee, only a general information notice in hospital structures is displayed. All data were fully anonymized before analyses.

### Data collection

For each case, the registry investigators collected every week data on clinical, laboratory, electrocardiographic variables, risk factors (hypertension, dyslipidemia, diabetes, and smoking status) and cardiovascular or non-cardiovascular comorbidities from the patient’s medical records.

Electrocardiograms (ECGs) were coded as (i) ‘ST+’ for the occurrence of a new Q-wave and/or occurrence of a new left bundle-branch block (LBBB) or the occurrence or presence of ST elevation; (ii) ‘non-ST+’ for other repolarization abnormalities (such as a negative T wave) and normal ECGs; (iii) ‘not classifiable’ for patients with a pacemaker, interventricular block, complete atrioventricular block, Wolf-Parkinson-White syndrome, ventricular fibrillation, ventricular rhythm, pre-existing LBBB, or right bundle-branch block, or (iv) ‘missing’ when the data were missing.

The left ventricular ejection fraction (LVEF) was classified (regardless of the measurement method) as ‘normal’ if it was above 50%, ‘moderately altered’ if it was between 35% and 50%, and ‘altered’ if it was below 35%. If several LVEF measurements were available, only the value recorded nearest to the time of hospital discharge was kept.

Treatments administered before and during the hospital stay and upon discharge (including revascularization therapies like angioplasty, fibrinolysis, and coronary bypass) were also documented.

With regard to the patient’s symptoms, ‘typical symptoms’ were defined as chest pain lasting more than 20 minutes or crescendo angina, and ‘major symptoms’ were defined as resuscitated cardiac arrest, acute pulmonary oedema, or cardiogenic shock.

Troponin values are looked for in the medical records of the patients. If a rise and/or a decrease in troponin values is described, only the highest troponin value of the peak is recorded and the value is coded as positive (defined as a serum troponin level greater than or equal to twice the laboratory’s normal upper limit), negative, or equivocal.

The types of ACS were defined as follows [14]: STEMI corresponded to an ST+ ECG and a positive troponin assay; NSTEMI corresponded to a non-ST+ ECG and a positive troponin assay; UA corresponded to a non-ST+ ECG and a negative troponin assay; “other” events corresponded to patients who had an acute coronary syndrome with an ST+ ECG but with equivocal or normal troponins (11%) or patients for whom the ECG data was unclassifiable or was missing (36%), or the troponin assay was missing (44%) or both ECG and troponines were missing (9%).

### Survival status

Survival 28 days and 1 year after the event was documented whenever possible. Vital status and causes of death were checked (i) in the hospital’s medical records, (ii) with the patient’s general practitioner, (iii) in the city registry office and (iv) in the MONICA registry database.

### Statistical analyses

After having validated the year 2017 in the registry, the statistical analyses were performed. All the study variables were categorical and are presented as the frequency (percentage). Groups were compared using a chi-squared test or (for small sample sizes) Fisher’s exact test. All analyses were adjusted for age and sex using a generalized linear model, a cumulative link model or multinomial logistic regression, as appropriate. Survival was analyzed using the Kaplan-Meier method and (when valid) the log-rank test. Adjusted Cox models were performed to assess mortality. All tests were two-sided, and the threshold for statistical significance was set to p<0.05. Analyses were performed using RStudio (version 1.2.1335).

## Results

The characteristics of hospitalized patients are summarized in Table 1. When considering the 1,459 patients hospitalized for an ACS, there were 1,028 (70%) incident cases and 431 (30%) recurrent cases. There was no significant difference in sex ratio between incident and recurrent cases (p = 0.15). Compared with patients with an incident ACS, patients with a recurrent ACS were older, more likely to present with UA (6% vs. 18%, respectively) or NSTEMI (38% vs. 44%, respectively) and less likely to present with STEMI (48% vs. 23%, respectively). Patients with a recurrent ACS were less likely to show typical symptoms (63%) than incident cases (72%); however, there was no significant intergroup difference in the frequency of major symptoms. The proportion of patients with an altered LVEF was higher for recurrent ACS.

**Table 1:** Main characteristics of patients admitted to hospital for an incident or recurrent acute coronary syndrome.

	N (%)	Incident cases	Recurrent cases	p	p*
	1,459	1,028 (70%)	431 (30%)		
Men	1,129 (77%)	787 (77%)	342 (79%)	0.24	0.15
35–44 years of age	92 (6%)	82 (8%)	10 (2%)	**<0.0001**	**<0.0001**
45–54 years of age	319 (22%)	252 (25%)	67 (16%)		
55–64 years of age	475 (33%)	359 (35%)	116 (27%)		
65–74 years of age	573 (39%)	335 (33%)	238 (55%)		
STEMI	589 (40%)	492 (48%)	97 (23%)	**<0.0001**	**<0.0001**
NSTEMI	576 (39%)	388 (38%)	188 (44%)		
Unstable angina	143 (10%)	64 (6%)	79 (18%)		
Other events	151 (10%)	84 (8%)	67 (16%)		
Typical symptoms	1,006 (69%)	734 (72%)	272 (63%)	**0.005**	**0.045**
Major symptoms:	188 (13%)	128 (12%)	60 (14%)	0.46	0.89
• resuscitated cardiac arrest	103 (7%)	78 (8%)	25 (6%)	0.22	0.3
• acute pulmonary edema	67 (5%)	36 (4%)	31 (7%)	**0.002**	0.051
• cardiogenic shock	87 (6%)	63 (6%)	24 (6%)	0.68	0.38
Normal LVEF	970 (66%)	737 (72%)	233 (54%)	**<0.0001**	**<0.0001**
Moderately altered LVEF	266 (18%)	180 (18%)	86 (20%)		
Altered LVEF	83 (6%)	46 (4%)	37 (9%)		
Missing LVEF	140 (10%)	65 (6%)	75 (17%)		
Deceased at 28 days	96 (7%)	56 (5%)	40 (9%)	**0.007**	0.1
Deceased at 1 year	131 (9%)	75 (8%)	56 (14%)	**0.0004**	**0.023**

STEMI: ST-segment elevation myocardial infarction; NSTEMI: Non-ST-segment elevation myocardial infarction; LVEF: Left ventricular ejection fraction.

*Adjusted for age and sex.

Significant p values are indicated in bold type.

### Risk factors and comorbidities

Concerning smoking status, recurrent cases were more likely to be former smokers than incident cases (39% vs. 23%, respectively) and less likely to be current smokers (33% vs. 49%, respectively) (Table 2). The prevalence of hypertension, dyslipidemia, obesity, and especially that of diabetes mellitus and complicated diabetes was higher among recurrent cases than among incident cases. Comorbidities such as a history of stroke (p = 0.0002), peripheral artery occlusive disease (p<0.0001), aortic aneurysm (p = 0.038), and renal failure (p<0.0001) were also significantly more common along recurrent cases.

**Table 2:** Risk factors and comorbidities in patients admitted to hospital for an incident or recurrent acute coronary syndrome.

	N = 1459	Incident cases	Recurrent cases	p	p*
		1028 (70%)	431 (30%)		
Never smoker	373 (28%)	266 (28%)	107 (28%)	**<0.0001**	**0.0005**
Current smoker	595 (44%)	467 (49%)	128 (33%)		
Former smoker	373 (28%)	222 (23%)	151 (39%)		
Hypertension	735 (51%)	448 (44%)	287 (67%)	**<0.0001**	**<0.0001**
Dyslipidemia	742 (51%)	422 (41%)	320 (75%)	**<0.0001**	**<0.0001**
Diabetes mellitus	401 (28%)	202 (20%)	199 (46%)	**<0.0001**	**<0.0001**
Complicated diabetes	86 (6%)	35 (3%)	51 (12%)	**<0.0001**	**<0.0001**
Obesity	390 (28%)	250 (25%)	140 (34%)	**0.001**	**0.0004**
Previous transient ischemic attack	22 (2%)	11 (1%)	11 (3%)	**0.03**	0.15
Previous stroke	66 (5%)	29 (3%)	37 (9%)	**<0.0001**	**0.0002**
Peripheral artery occlusive disease	132 (9%)	43 (4%)	89 (21%)	**<0.0001**	**<0.0001**
Aortic aneurysm	22 (2%)	9 (1%)	13 (3%)	**0.002**	**0.038**
Chronic obstructive pulmonary disease	82 (6%)	47 (5%)	35 (8%)	**0.007**	0.076
Renal failure	102 (7%)	40 (4%)	62 (14%)	**<0.0001**	**<0.0001**

*Adjusted for age and sex. Significant p values are indicated in bold type.

### Medical treatments before the hospital stay and upon discharge

**Fig 1:**
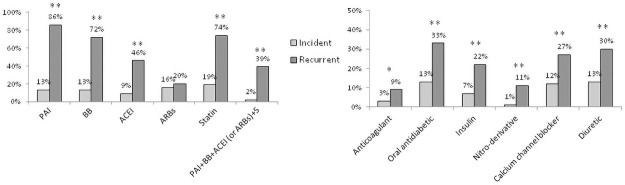
Drug treatments before hospital admission in patients with an incident or recurrent acute coronary syndrome. *p<0.001, **p<0.0001, after adjustment for sex and age. PAI: Platelet aggregation inhibitor; BB: Beta-blocker; ACEI: Angiotensin-converting-enzyme inhibitor; ARBs: Angiotensin receptor blockers; S: Statin.

**Fig 2:**
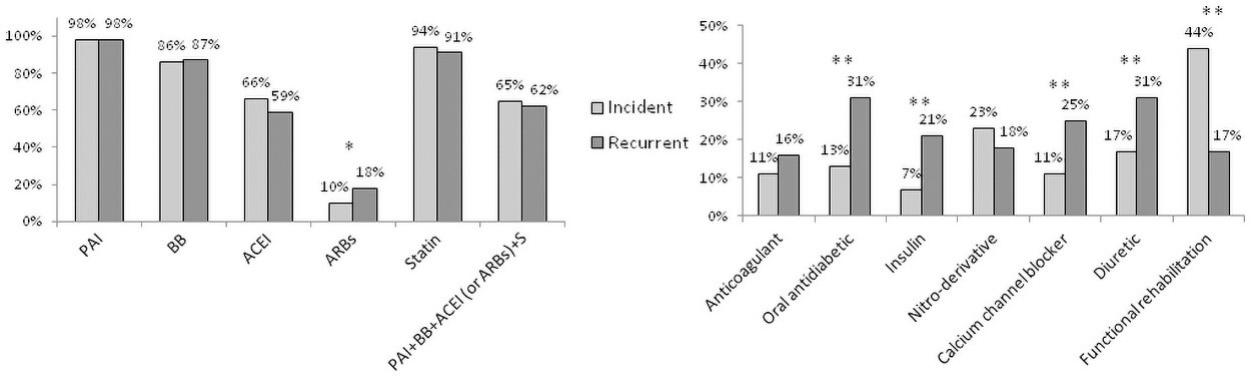
Drug treatments upon hospital discharge in patients with an incident or recurrent acute coronary syndrome. *p<0.05, **p<0.0001, after adjustment for sex and age. PAI: Platelet aggregation inhibitor; BB: Beta-blocker; ACEI: Angiotensin-converting-enzyme inhibitor; ARBs: Angiotensin receptor blockers; S: Statin.

On admission to hospital, platelet aggregation inhibitors, beta-blockers, ACE inhibitors and statins were administered to respectively 86%, 72%, 46% and 74% of the recurrent cases, whereas, as expected, they were dispensed to less than 20% of the incident cases (Fig 1). These proportions were higher upon discharge for recurrent cases: 98%, 87%, 59%, and 91% for platelet aggregation inhibitors, beta-blockers, ACE inhibitors, and statins, respectively. At discharge, there were no significant differences in the recommended treatments for secondary prevention (i.e. beta-blockers/platelet aggregation inhibitors/statins/ACE inhibitors) between incident and recurrent cases (Fig 2).

Other treatments (such as insulin, orally administered antidiabetics and diuretic drugs) were more frequently prescribed in recurrent than in incident cases before the hospital stay (Fig 1). This is consistent with a higher prevalence of diabetes, hypertension, and renal failure in patients with a recurrent ACS (Table 2). Similar differences persisted between the two patient groups at discharge (Fig 2). Of note, recurrent patients with diabetes were treated with insulin at least twice as often as incident patients with diabetes, both before the hospital stay and at discharge. Last, upon discharge and after adjustment for sex and age, functional rehabilitation was more frequently prescribed for incident cases than for recurrent cases (44% vs. 17%, respectively) (Fig 2).

### Revascularization therapy

When considering STEMI/UA, there was no significant difference between incident and recurrent cases with regard to the incidence of revascularization therapy during the hospital stay and at discharge (Table 3). In contrast, in NSTEMI, angioplasty was significantly less frequent in recurrent cases than in incident cases during the hospital stay (63% vs 74%, respectively, p = 0.015) and upon discharge (7% vs 14%, respectively, p = 0.038).

**Table 3:** Reperfusion therapy during the hospital stay and at discharge in patients admitted to hospital for an incident or recurrent acute coronary syndrome, according to the type of event (STEMI, NSTEMI, UA, and other events).

		**STEMI**					**NSTEMI**				
		N = 589	Incident cases	Recurrent cases	p	p**	N = 576	Incident cases	Recurrent cases	p	p**
			492 (84%)	97 (16%)				388 (67%)	188 (33%)		
**During the hospital stay**	Angioplasty	533 (90%)	445 (90%)	88 (91%)	0.9	1	404 (70%)	286 (74%)	118 (63%)	**0.007**	**0.015**
	Fibrinolysis	13 (2%)	12 (2%)	1 (1%)	0.7	0.5	3 (1%)	2 (1%)	1 (1%)	1	0.81
	Coronary bypass	9 (2%)	8 (2%)	1 (1%)	1	0.8	29 (5%)	21 (5%)	8 (4%)	0.69	0.14
	All revascularizations	542 (92%)	453 (92%)	89 (92%)	0.9	0.9	431 (75%)	305 (79%)	126 (67%)	**0.002**	**0.001**
**Upon discharge** *	Angioplasty	62 (11%)	49 (10%)	13 (13%)	0.4	0.6	65 (12%)	52 (14%)	13 (7%)	**0.029**	**0.038**
	Coronary bypass	8 (1%)	7 (1%)	1 (1%)	1	0.8	18 (3%)	10 (3%)	8 (4%)	0.3	0.47
		**Unstable Angina**					**Other**				
		N = 143	Incident cases	Recurrent cases	p	p**	N = 151	Incident cases	Recurrent cases	p	p**
			64 (45%)	79 (55%)				84 (56%)	67 (44%)		
**During the hospital stay**	Angioplasty	99 (69%)	45 (70%)	54 (68%)	0.8	0.7	95 (63%)	60 (71%)	35 (52%)	**0.015**	**0.018**
	Fibrinolysis	-	-	-	-	-	2 (1%)	1 (1%)	1 (2%)	1	0.78
	Coronary bypass	5 (3%)	3 (5%)	2 (3%)	0.7	0.4	4 (3%)	3 (4%)	1 (1%)	0.63	0.31
	All revascularizations	102 (71%)	47 (73%)	55 (70%)	0.6	0.5	100 (67%)	63 (75%)	37 (56%)	**0.015**	**0.013**
**Upon discharge** *	Angioplasty	11 (8%)	7 (11%)	4 (5%)	0.2	0.2	19 (15%)	10 (14%)	9 (17%)	0.62	0.59
	Coronary bypass	5 (4%)	2 (3%)	3 (4%)	1	0.8	6 (5%)	4 (5%)	2 (4%)	1	0.92

The “all reperfusion therapies” group included fibrinolysis, angioplasty and coronary bypass surgery. STEMI: ST-segment elevation myocardial infarction; NSTEMI: Non-ST-segment elevation myocardial infarction.

*As a percentage of patients who survived the acute event.

**Adjusted for age and sex. Significant p values are indicated in bold type.

### Survival outcome

**Fig 3:**
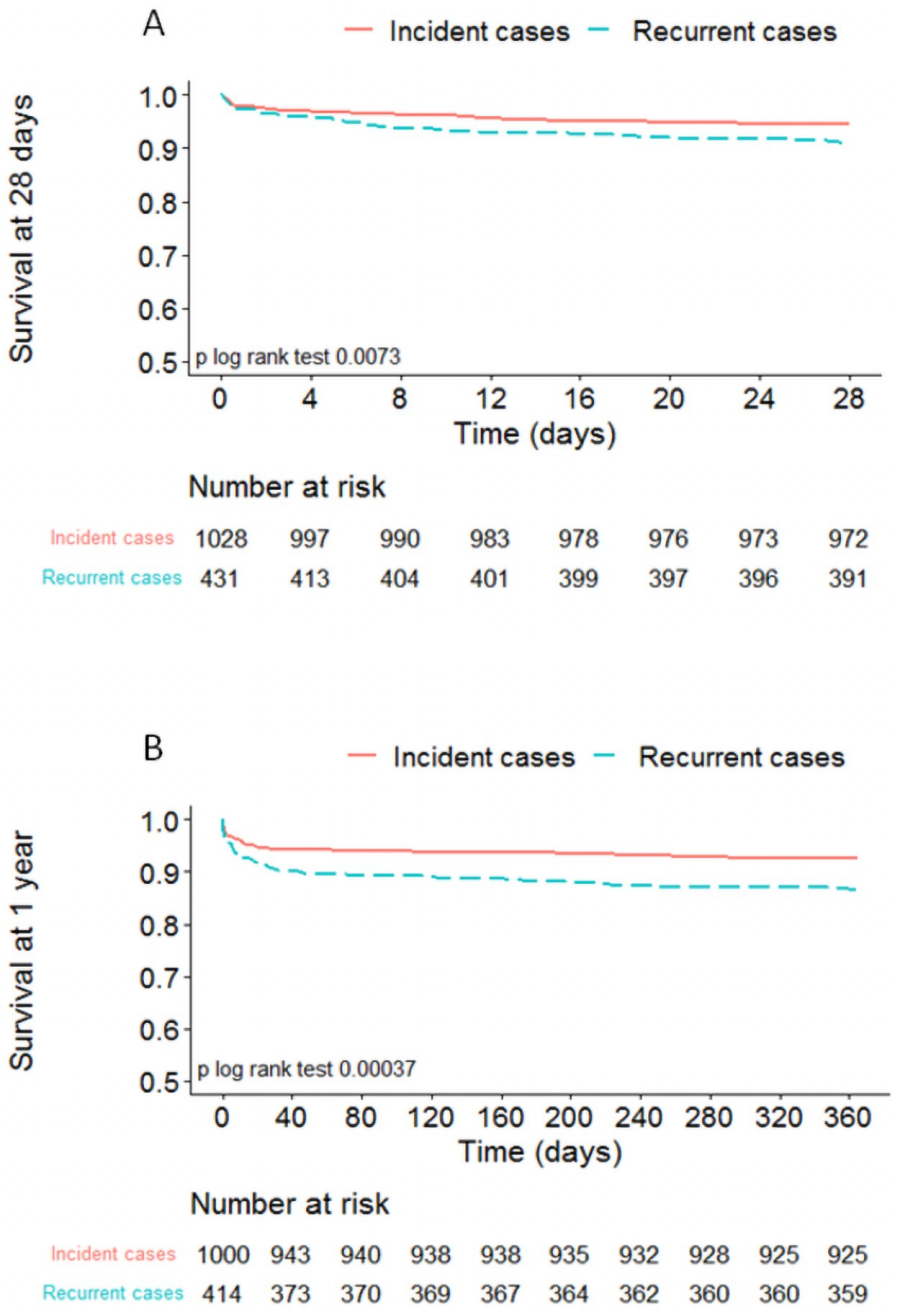
28-day and 1-year survival curves. A. 28-day survival curves. B. 1-year survival curves.

The 28-day and 1-year survival rates were both lower (p = 0.0073 and p = 0.00037, respectively) in recurrent than in incident patients (Fig 3). The mortality rates were 9% vs 5% at 28 days and 14% vs 8% at 1 year, in recurrent and in incident patients, respectively (Table 1). However, the adjusted hazard ratios [95% CI] for the 28-day and 1-year mortality were no longer different (after adjustment for age, sex, region, diagnosis category and LVEF) between the two groups (0.71 [0.45–1.12] and 0.87 [0.59–1.28], respectively).

## Discussion

In this ancillary study of the French MONICA CHD registries all consecutive patients who had been hospitalized for an ACS were investigated for symptoms, biology, risk factors, acute-phase clinical care procedures and treatments. STEMI were more common than any other ACS subtypes among incident patients, whereas NSTEMI and UA predominated among recurrent cases. Patients with a recurrent ACS were more likely to have comorbidities, risk factors and CHD treatments prior to admission than incident ACS. The proportion of revascularization therapy was similar between STEMI and UA incident and recurrent patients but was lower in recurrent than incident NSTEMI patients. Finally, the incident and recurrent cases did not differ with regard to secondary prevention treatments at discharge.

### Categories of ACS

During the observation period, the prevalence of incident and recurrent ACS was 70% and 30%, respectively. When considering only incident events, the prevalence of STEMI, NSTEMI and UA were respectively 48%, 38% and 6%; these proportions differed for recurrent patients reaching 23%, 44% and 18%, respectively. These values are in line with literature reports of a higher prevalence of NSTEMI among recurrent patients than among incident patients [2,3,5]. This observation might be due to greater awareness of the symptoms and risks of ACS among patients who have already experienced an ACS event; these patients might reach the hospital sooner (i.e. before they develop a STEMI) than incident patients do. In agreement with this hypothesis, other studies have reported shorter time intervals between symptom onset and hospital admission in patients with recurrent as compared with incident events [6,15]. Furthermore, secondary prevention with drugs might attenuate the severity of ACS, resulting in recurrent NSTEMI and UA rather than a STEMI. Plaque instability and inflammation status can also play a role. Lastly, the presence of coronary lesions in patient with history of coronary heart disease can lead to angina pectoris, changes on the ECG, troponin elevation resulting in a more frequent diagnosis of NSTEMI or UA for recurrent ACS. This hypothesis is supported by the observation that recurrent cases were less likely to present typical symptoms than incident cases. The practical implications of this observation is that after a first ACS, patients should be informed that the clinical presentation of recurrent infarction might differ from the initial infarction [16].

### Mortality at 1 year

Our results showed that the mortality rate at 1 year was higher among recurrent cases than among incident cases (14% vs. 8%, respectively), independently of age and sex. The same trend was found for mortality at 28 days (9% vs. 5%, respectively), indicating that the difference in prognosis between recurrent and incident events arises soon after the acute event. Similar results have been reported in other studies [3–5]. However, the rates in these studies were not always adjusted for confounding variables such as age, sex, comorbidities, and other relevant variables. In the Cox multivariate analysis, the difference in 1-year mortality between recurrent and incident patients was no longer statistically significant (after adjustment for age, sex, region, diagnosis category and LVEF) suggesting that a poorer risk factors profile, including older age and altered cardiac function, explain the worse prognosis in recurrent patients [17].

### Risk factors and comorbidities

The prevalence of risk factors and comorbidities was substantially higher in recurrent patients than in incident patients which might have contributed to the recurrence [18]. In addition, the recurrent patients are more likely to have undergone screening for cardiovascular risk factors resulting in higher prevalence of these risk factors. With regard to smoking, and although the prevalence of current smoking was lower among recurrent patients than among incident patients in our study, an unhealthily high proportion (33%) of recurrent patients were still smoking. Given that smoking cessation in patients with CHD is associated with a lower mortality rate [19], intense efforts must be undertaken to achieve smoking cessation.

Among patients with ACS, those with diabetes mellitus are at particularly high risk of recurrent cardiovascular events and premature death. In pooled 10 European registries, diabetes mellitus was associated with higher all-cause death, higher cardiovascular death and major bleeding [20]. In an Italian registry with around 2500 patients, it was shown that hyperglycemic patients with obstructive acute MI had higher inflammatory markers and larger infarct sizes compared to normoglycemic ones [21]. In addition, admission stress-hyperglycemia predicted higher in-hospital and long-term (3 year) mortality, regardless of diabetes, in both patients with obstructive or non-obstructive coronary arteries [22].

Altogether, these data suggest that secondary prevention is suboptimal, as it has been shown in several EUROASPIRE studies over the last 20 years in Europe [23,24].

### Treatments

On admission to hospital, the proportions of patients taking various cardiovascular medications (platelet aggregation inhibitors, beta-blockers, ACE inhibitors, and statins) were higher among recurrent cases than among incident patients—although the overall values appeared to be suboptimal. Similar observations for recurrent cases have been reported in other settings [2,5]. Many factors may contribute to the failure of suboptimal medical treatment after a first ACS, including adverse drug reactions, poor treatment adherence, and poor coordination between specialists and family physicians. The fact that incident and recurrent patients had similar and apparently optimal treatment profiles at discharge from hospital shows that the level of secondary prevention is not influenced by MI history and that it is possible to achieve the required level of treatment among recurrent patients. Finally, the proportion of patients who were prescribed functional rehabilitation was more than twice as high among incident cases than among recurrent cases (44% vs. 17%, respectively). This difference might be explained by the fact that some of the recurrent patients had already undergone functional rehabilitation after their first ACS. Yet as recurrent patients have more co-morbidities they might benefit from further rehabilitation program after their recurrent event.

### Revascularization therapy

The prevalence of revascularization therapy during the hospital stay and at discharge was similar for incident and recurrent STEMI and UA patients—suggesting that revascularization is not influenced by previous status among these ACS subtypes. In contrast, angioplasty among patients with NSTEMI was less frequent among recurrent cases than among incident cases (63% vs. 74% during the hospital stay, and 7% vs. 14% at discharge, respectively). These observations are consistent with a study in which prior MI did not influence the revascularization approach for STEMI patients but was associated with less frequent use of angiography and revascularization for NSTEMI patients [2]. This difference might be due to a greater prevalence of complex coronary lesions in recurrent NSTEMI patients.

### Limitations and strengths

Our study had some limitations. First, although data were collected in 2015–2016 and survival monitoring extended until 2017, these are the most-updated direct comparisons of incident and recurrent ACS to date, especially with regard to the lethality which has decreased over the past decade. Second, with regard to the lower proportion of recurrent patients prescribed functional rehabilitation, we could not determine whether or not they had already participated in this activity after their incident event. Third, given the relatively low number of recurrent cases recorded (n = 431), some of our analyses might have lacked statistical power. Fourth, it would have been interesting to analyse the impact of the site of myocardial infarction on mortality but this variable was not collected in the registry. Lastly, as the age limit for inclusion in the registry was 74, our results cannot be extrapolated to older age groups. The present study had several methodological strengths. It included exhaustive 6-month data from three French morbidity registries. Data were recorded in accordance with the MONICA registry’s methodology by trained investigators able to adjudicate all coronary events. The MONICA registries cover all public and private hospitals and all hospitalized patients—regardless of the admission pathway. The registries are reliable, and the coverage in each region is close to 100%.

### Conclusion

Our results show that in the areas of France covered by our registries, around 30% of patients hospitalized for an ACS had experienced a recurrent event. The higher 1-year mortality rate observed among recurrent cases (relative to incident cases) was explained by older age, comorbidities and worse cardiac function—emphasizing the need to reinforce secondary prevention after an ACS and thus act on persistent risk factors.
